# Marine Invertebrate Peptides: Antimicrobial Peptides

**DOI:** 10.3389/fmicb.2021.785085

**Published:** 2021-12-16

**Authors:** Ran Wu, Jiri Patocka, Eugenie Nepovimova, Patrik Oleksak, Martin Valis, Wenda Wu, Kamil Kuca

**Affiliations:** ^1^MOE Joint International Research Laboratory of Animal Health and Food Safety, College of Veterinary Medicine, Nanjing Agricultural University, Nanjing, China; ^2^Department of Radiology and Toxicology, Faculty of Health and Social Studies, University of South Bohemia in České Budějovice, České Budějovice, Czechia; ^3^Biomedical Research Centre, University Hospital Hradec Králové, Hradec Králové, Czechia; ^4^Department of Chemistry, Faculty of Science, University of Hradec Králové, Hradec Králové, Czechia; ^5^Department of Neurology, Faculty of Medicine, University Hospital Hradec Králové, Charles University, Hradec Králové, Czechia

**Keywords:** antimicrobial peptides, marine invertebrate, activity, mechanism, marine

## Abstract

Antimicrobial peptides are an important component of many organisms’ innate immune system, with a good inhibitory or killing effect against the invading pathogens. As a type of biological polypeptide with natural immune activities, antimicrobial peptides have a broad spectrum of antibacterial, antiviral, and antitumor activities. Nevertheless, these peptides cause no harm to the organisms themselves. Compared with traditional antibiotics, antimicrobial peptides have the advantage of not producing drug resistance and have a unique antibacterial mechanism, which has attracted widespread attention. In this study, marine invertebrates were classified into arthropods, annelids, mollusks, cnidarians, and tunicata. We then analyzed the types, sources and antimicrobial activities of the antimicrobial peptides in each group. We also reviewed the immune mechanism from three aspects: membrane-targeted direct killing effects, non-membrane targeting effects and immunomodulatory effects. Finally, we discussed their applications and the existing problems facing antimicrobial peptides in actual production. The results are expected to provide theoretical support for future research and applications of antimicrobial peptides in marine invertebrates.

## Introduction

Antimicrobial peptides (AMP) are small positively charged peptides that promote the innate defense mechanism by targeting the negatively charged membranes of microorganisms (Brogden, 2005). After encountering the microbial cell envelope, AMP will be embedded in the hydrophobic area of the lipid membrane, resulting in membrane instability and ultimately cell death (Joo et al., 2016). Since the first animal AMPs were discovered in silkworms, insect AMPs, especially *Drosophila melanogaster* AMPs, have attracted great attention (Steiner et al., 1981). There are currently seven well-defined inducible AMP families in *Drosophila melanogaster*. The activities of these AMPs have been determined *in vitro* or inferred by comparison with homologous peptides of other insects: metchnikowin and drosophilin show antifungal activity (Levashina et al., 1995); drosocin, diptericins and attacins mainly show antibacterial activity (Kragol et al., 2001). Defensin and cecropins have antibacterial and some antifungal activities (Tzou et al., 2002). In the systemic response after microbial recognition, these AMPs are regulated by the Toll and Imd NF-κB signaling pathways (Clemmons et al., 2015). Therefore, AMP is often used as a readout to monitor the activity of these immune pathways.

Marine invertebrates do not have a memory-type acquired immunity that is built on T-lymphocyte subsets and clonally derived immunoglobulin (Relf et al., 1999). Cellular immunity in marine invertebrates originates from defense reactions, which include hemocyte-mediated modules, encapsulation and phagocytosis. The cellular part of the immunity in question is hemocyte-mediated motile cells that phagocytize microbes and exude soluble antimicrobial and cytotoxic elements from the hemolymph (Mitta et al., 1999a). This is different from other species such as insects. Instead, insects depend heavily on challenge-induced synthesis of antimicrobial peptides through fat in the body and use exclusion, via a tough exoskeleton, as their major antimicrobial defense (Iwanaga and Kawabata, 1998). Insects are different because they produce both humoral and cellular reactions. Cellular immunity in insects has to do with phagocytosis, nodule formulation, and encapsulation (Wright, 1981). The cellular component of marine invertebrate immunity is mediated by hemocytes, motile cells that phagocytize microbes and secrete soluble antimicrobial as well as cytotoxic substances into the hemolymph (Mitta et al., 2000a). Humoral immunity in marine invertebrates is marked by antimicrobial agents that typically already exist within the plasma and blood cells. Marine invertebrates’ ability to survive in the given environment is evidence that they have a robust and effective immune system (Menzel et al., 2002).

The circulating hemolymph in marine invertebrates contains biologically active substances such as complement, lectins, clotting factors, and antimicrobial peptides (Miyata et al., 1989). Recently, with the extensive use of antibiotics, multi-drug-resistant pathogens have appeared, and bacteria that can no longer be controlled by conventional medicine have been on the rise. This has caused serious health and medical issues. To address this problem, creating new antibacterial agents that can deal with the issue is imperative. Endogenous antimicrobial peptides may prove useful in helping develop a solution. This is because of their extremely selective toxicities and broad antimicrobial spectra, and their ability to inhibit bacteria when they look to develop resistance to the peptides in question (Hancock and Scott, 2000).

Marine invertebrates do not have immunoglobulin, complement and other specific immune system components, but they can survive in the competitive complex marine environment. They must have evolved a complete system of immune defenses. When microbes invade, they activate the immune mechanism to protect themselves (Kang et al., 2019; Contreras et al., 2020). Studies have shown that antimicrobial peptides are a crucial aspect of immune defense. When it comes to the invertebrates in question, the peptides play an important role in immune response. Antimicrobial peptides isolated from marine organisms are defensive peptides produced by the body to resist the invasion of pathogenic microorganisms. As a natural product, these peptides have a broad spectrum of antimicrobial properties, so they can kill both Gram-negative and Gram-positive bacteria. They have a novel and unique structure, and few side effects. Moreover, they not only have a strong antimicrobial effect, but are also involved in immune regulation, antitumor cells and other function (Agarwal and Gabrani, 2020; Chen and Lu, 2020; Mercer et al., 2020). The peptides are rich in lysine or arginine, which gives the antibacterial peptide a positive charge, and contain hydrophobic amino acids to form a hydrophobic or amphiphilic structure (Chaturvedi et al., 2020). These peptides are smaller than 10 kDa in terms of their mass. However, antimicrobial properties have been observed in the peptides and produced a rapid and immediate reaction to microorganisms that were attempting to invade the system (Boman, 1995; Bartlett et al., 2002). Marine invertebrates are different from vertebrates in terms of their immune system. The former is marked by somatic gene rearranging, clonal expansion and selection, and a discriminative ability that involves several factors, including lymphocytes.

Due to the lack of specific antibodies, marine invertebrates mainly rely on the non-specific immune system. The system comprises mucous membranes, the exoskeleton, and cell activities in blood or body cavity fluid and humoral factors to protect themselves. The body fluid is the main defense site. In this study, the antimicrobial peptides in body fluid factors in invertebrates were reviewed to provide a reference for marine invertebrate culture and antimicrobial peptide research.

## Marine Invertebrates’ Peptides

### Marine Organisms and Marine Invertebrates

As the largest ecosystem on the earth, the ocean is extremely rich in organisms. Marine organisms include microbes, plants, vertebrates, and invertebrates. All these organisms can produce a large set of structurally varying products of an antimicrobial nature (Ely et al., 2004). Some of them come from marine animals and others have been found to originate from microbes linked to spores (Mayer and Hamann, 2004). The range runs from tiny organic compounds to macromolecules. Some are unique in terms of their structure, whereas others come from other compound classes including proteins, alkaloids, and peptides. Antimicrobial peptides are the main component of the innate immune defense system in marine invertebrates. Some antibacterial agents are more active against Gram-positive bacteria, while others have greater efficacy against Gram-negative bacteria. Some are active against both Gram-positive and Gram-negative bacteria (Shaw et al., 1976). The mechanism of antibacterial action of a large amount of chemically identified antibacterial substances, such as their synergy with typically deployed antibiotics, and the question of likely mammalian and tissue toxicities are waiting for clarification. The structural elements that are significant for antibacterial activity have been identified in only a handful of studies. Examples of the efficacy of antibacterial agents in animal models of bacterial infection are highly desirable. The structural aspects of the active substances with supposed antibacterial activity found in aqueous and organic extracts from marine organisms would prove useful.

Around 71% of the world’s surface is covered by water. The expanse of the ocean contains half of the world’s biodiversity, and marine macrofauna alone comprise up to 107 species (De Vries and Hall, 1994). Invertebrates represent more than 50% of the marine species documented in marine waters, including jellyfish, sea anemones, lobsters, crabs, sea stars, mollusks, octopuses, etc. They occur in large numbers throughout estuaries, coastal coves and deep seas. They are also responsible for producing a wide range of biologically active compounds, among which a significant group consists of peptides with antimicrobial activity. These organisms rely solely on innate immune systems as a means of defense. This may prove to be a very useful element when it comes to developing new antimicrobial compounds. Due to the existence of wide biodiversity and many unique life forms, the ocean has become a treasure house for searching for and exploring many bioactive substances, including antimicrobial peptides.

### Marine Invertebrates’ Antimicrobial Peptides

Invertebrates are the main source of antimicrobial peptides in marine organisms. These peptides are essential elements for marine invertebrates and their immune defense (Tincu and Taylor, 2004). They are constitutively expressed and rapidly induced to modulate the immunoreactions involved in defense against pathogenic microorganisms. In this context, antimicrobial peptides represent the major humoral defense system against infection. Marine invertebrates are constantly under an enormous microbial challenge from the ocean environment. Over the past two decades, a number of antimicrobial peptides have been isolated from marine invertebrates, including cnidarians, mollusks, annelids, arthropods, and tunicata (Table 1; Otero-González et al., 2010). Antimicrobial peptides hold great promise, because their initial interaction with microbes is executed through lipid binding (Sperstad et al., 2011).

**TABLE 1:** Overview of the gene-encoded AMPs from marine invertebrates, including some key features.

Phyla	AMP types	Origin	Size	Cys	Activity spectrum^a^
Annelida	Arenicins	Coelomocytes	21 aa	2	G^+^, G^–^, F
	Perinerin	Homogenate	51 aa	4	G^+^, G^–^, F
	Hedistin	Coelomocytes	22 aa	0	G^+^, G^–^, F
Mollusca	Defensin	Hemocytes	39–43 aa	6–8	G^+^, G^–^
	Mytilin	Hemocytes	32–34 aa	8	G^+^, G^–^, F
	Myticin	Hemocytes	40 aa	8	G^+^, (G^–^, F)
	Mytimycin	Hemocytes	54 aa	12	F
Arthropoda	Tachyplesins	Hemocytes	17 aa	4	G^+^, G^–^, F
	Polyphemusins	Hemocytes	18 aa	4	G^+^, G^–^, F
	Penaeidins	Hemocytes	47–67 aa	6	G^+^, (G^–^), F
Cnidaria	Aurelin	Ectoplasm	40 aa	6	G^+^, G^–b^
Tunicata	Styelins	Hemocytes	31–32 aa	0	G^+^, G^–b^
	Clavanins	Hemocytes	23 aa	0	G^+^, G^–^, F
	Clavaspirin	Pharyngeal	23 aa	0	G^+^, G^–^, F
	Plicatamide	Hemocytes	26 aa	0	G^+^, G^–b^
	Halocyamine	Hemocytes	4 aa	0	G^+^, G^–^, F
	Dicynthaurin^c^	Hemocytes	30 aa	2	G^+^, G^–^
	Halocidin^c^	Hemocytes	18 aa	2	G^+^, G^–^, F^d^

*^a^G^+^, Gram-positive bacteria; G^–^, Gram-negative bacteria; F, fungi. Brackets indicate microorganisms with considerably lower susceptibility to the AMP.*

*^b^The AMP has not been screened against all groups of microorganisms included in this table.*

*^c^Dimeric AMPs.*

*^d^A synthetic analog of halocidin showed antifungal activity.*

### Annelida

Annelids are made up of wormlike animals such as earthworms, leeches, and polychaetes. The antimicrobial peptides isolated from this phylum includes arenicins, perinerin, and hedistin.

#### Arenicins

Two 21-residue antimicrobial peptides, arenicin-1 and arenicin-2 (molecular masses: 2758.3 and 2772.3), were purified from coelomocytes of the marine polychaeta *Arenicola marina* (lugworm). Each arenicin has one disulfide bond (Cys3–Cys20). Arenicins have no structural similarity to any previously identified antimicrobial peptides (Ovchinnikova et al., 2004). The peptides have a high positive charge (+6) and a highly twisted β-hairpin in water, or planar β-structural dimers in anionic liposomes and detergent micelles. They display a wide spectrum of antimicrobial activities against fungi and bacteria (Sychev et al., 2018). Arenicin-1 can be used in combination with antibiotics such as ampicillin to inhibit the growth of bacteria and achieve better antibacterial effects (Choi and Lee, 2012).

Arenicin-1: RWCVYAYVRVRGVLVRYRRCW

Arenicin-2: RWCVYAYVRIRGVLVRYRRCW.

#### Perinerin

Perinerin, a 51-residue antimicrobial peptide with four cysteines, was purified from a homogenate of *Perinereis aibuhitensis*. The sequence is different from other peptides and is unique. The spiral-angle-helical structure shows obvious amphiphilic (lipophilic and hydrophilic) properties. It has antibacterial activities against Gram-positive and Gram-negative bacteria, and fungi (Pan et al., 2004).

#### Hedistin

Hedistin, isolated from the coelomocytes of *Nereis diversicolor*, has a broad spectrum of antimicrobial activity, including methicillin-resistant *Staphylococcus aureus* and *Vibrio alginolyticus* (Tasiemski et al., 2007).

### Mollusca

The mollusks mainly include clams, mussels, squid, octopuses, polyplacophorans, and gastropods. Mollusks are one of the most studied groups in terms of marine antimicrobial peptides. The number of mollusks is second only to that of arthropods, among which mussels have been the most thoroughly studied. Most of their antimicrobial peptides are cationic peptides rich in cysteine. According to the primary structure and the different types of disulfide bond, they can be divided into four kinds: defensin, mytilin, myticin, and mytimycin.

#### Defensins

Defensins are peptides identified by their cationic characteristics, small molecular mass and the strong presence of cysteine residues. This is one of the most diverse antimicrobial peptide groups within the invertebrates.

Pv-Def is defensin was found and catalogued in the Asian green mussel *Perna viridis*. The mature Pv-Def peptide contains 45 amino acids and six cysteines which formed three disulfide bonds at 27C1–54C4, 40C2–60C5, and 44C3–62C6. As is the case with a large portion of this family, mature Pv-Def peptides contain an α helix and two β strands (Wang et al., 2018).

Mgdefdg is a defensin from the digestive gland of the oyster *Magallana gigas*, whose shell can become as large as 18 cm long. The mature peptide has a cysteine-stabilized α-helix/β-sheet motif (CSαβ) and the consensus pattern C-X5-6-C-X3-C-X4-6-C-X3-4-C-X7-8-C-X-C-X2-C forming the potential disulfide linkages C1-C5, C2-C6, C3-C7, and C4-C8 in the predicted tertiary structure (Zhang et al., 2018).

RpdefB is defensin found and categorized from the manila clam *Ruditapes philippinarum*. The RpdefB peptide has 70 amino acids with a calculated molecular mass of 7.5 kDa and an isoelectric point of 8.16. The synthetic peptide RpdefB showed high antibacterial activity against the Gram-negative bacterium *Vibrio splendidus* (Yang et al., 2018).

#### Mytilins

Mytilins are the most diverse and abundant antimicrobial peptide family found in mussels. Mytilins have five subtypes: A, B, C, D, and G1. Mytilins are remarkably rich in cysteine residues with respect to their small size, suggesting that their three-dimensional structure is highly compact (Zhang et al., 2018). Mytilins A and B, which are cation cysteine-rich antimicrobial peptides, were isolated from *Mytilus edulis*. They showed no homology to known peptides in the peptide sequence database (Charlet et al., 1996). They showed activity against Gram-positive bacteria. Mytilin C, D, and G1 isoforms were isolated from *Mytilus galloprovincialis* and showed complementary antimicrobial properties (Mitta et al., 2000b). The five mytilin subtypes all have bactericidal activity, but their bactericidal effects are not the same. For example, D and G1 need 2∼6 h to completely kill *Micrococcus luteus*, while B and C only need 3∼10 min (Yang et al., 2012).

#### Myticins

Myticins contain eight conserved cysteine residues, including three subtypes: Myticins A, B, and C. Myticin A and Myticin B have different antibacterial activities. For example, Myticin B has moderate antibacterial activity against *Escherichia coli*, while Myticin A has no such activity (Mitta et al., 1999b). Myticin C has a high degree of molecular polymorphism; it is also the only peptide expressed in the larval stage of mussels. It has antiviral, developmental control and immunomodulatory activities (Balseiro et al., 2011).

#### Mytimycin

Furthermore, a novel antifungal peptide, mytimycin, was found and partially characterized in conjunction with defensins and mytilins from *Mytilus edulis*. Mytimycin has no homology to known peptide sequences in the protein database. This new antifungal peptide contains 12 conserved cysteine residues, and it inhibited the growth of *Neurospora crassa* and *Fusarium culmorum* (Charlet et al., 1996; Sonthi et al., 2012).

### Arthropoda

Arthropods among the most successful animals in terms of adaptation and survival. At present, a variety of antimicrobial peptides have been found in crustaceans (shrimp) and chelicerata (crab). Horseshoe crabs (*Tachypleus* spp.) are among the marine organisms with relatively high medicinal value. Tachyplesin antimicrobial peptides have been isolated from the horseshoe crab, including tachyplesin and polyphemusin. Penaeidins are the main antimicrobial peptides extracted from shrimps.

#### Tachyplesins

These are cationic peptides isolated from large hemocyte granules found in the horseshoe crab *Tachypleus tridentatus* (Nakamura et al., 1988). Based on two disulfide bridges between C3-C16 and C7-C12, the peptides consist of 17–18 residues with a C-terminal arginine α-amide and four cysteine residues (Muta et al., 1990).

**FIGURE 1:**
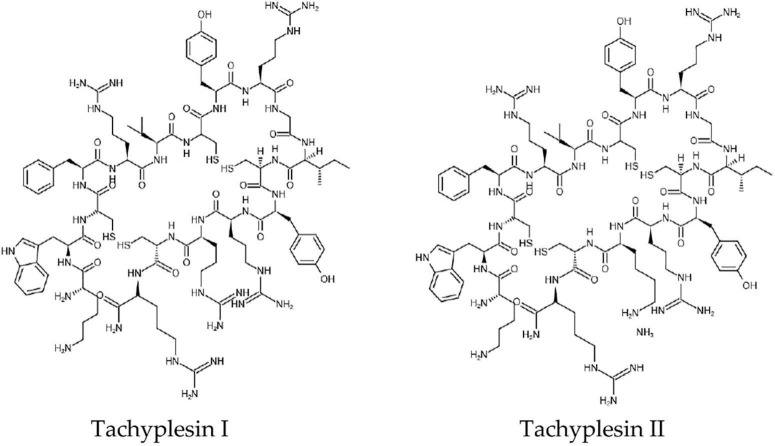
The chemical structures of Tachyplesin I and Tachyplesin II.

Tachyplesins are antimicrobial cationic 17-residue peptides. They were first isolated from hemocytes of the horseshoe crab (*Tachypleus tridentatus*) with a molecular weight of 2.36 kDa. Later, other analogs, including Tachyplesin I, Tachyplesin II, Tachyplesin III, Polyphemusin I, and Polyphemusin II were found (Xie et al., 2016) (Figure 1). These peptides are homologous. Tachyplesin I offers a wide spectrum when it comes to antimicrobial activity against Gram-negative bacteria, Gram-positive bacteria, fungi, and viruses (Li et al., 2017). The antitumor activity of Tachyplesin I has been shown in many tumor cells (Kuzmin et al., 2018).

#### Polyphemusins

These are some of the antimicrobial peptides found in hemocytes from American horseshoe crab, *Limulus polyphemus* (Hong et al., 2016). It is a peptide 18 amino acids long and is stabilized into an amphiphilic, antiparallel β-hairpin by two disulfide bridges. It has superior antimicrobial activity against Gram-negative and Gram-positive bacteria. With MICs less than 1 μg/mL, it has shown rapid killing effect within 5 min of administering treatment (Zhang et al., 2000).

Polyphemusin I RRWCFRVCYRGICYRKCR-CONH2

Polyphemusin II RRWCFRVCYRGICYRKCR-CONH2.

#### Penaeidins

Penaeidins are highly cationic peptides which were purified from the hemolymph of *Litopenaeus vannamei*. Penaeidins contain six cysteines, with sizes ranging from 47 to 67 amino acids (Tassanakajon et al., 2010). These comprise an N-terminal signal peptide sequence (SS) and a proline-rich region domain (PRD), followed by a C-terminal cysteine-rich domain (CRD) stabilized by three intramolecular disulfide cross-links (Destoumieux et al., 2000; Jiang et al., 2015). Penaeidins are different from every other defensin peptide that has been identified until now (Bachere et al., 2000). Although penaeidins have no activity against *Candida albicans* and *Saccharomyces cerevisiae*, they showed obvious resistance against filamentous fungi (Destoumieux et al., 1999). Penaeidins showed definite antibacterial activity against *Fusarium oxysporum*, and some Gram-positive bacteria and Gram-negative bacteria, such as *Vibrio* (Song and Lee, 1993).

### Cnidaria

Cnidarians are a group of aquatic carnivores that include hydra and jellyfish. Their antimicrobial peptides include aurelin and hydrolysin, but the latter has no antimicrobial activity.

Aurelin was purified from the mesoglea of a scyphoid jellyfish *Aurelia aurita* by preparative gel electrophoresis and RP-HPLC. Aurelin is a 40-residue antimicrobial peptide with a molecular mass of 4296.95 Da. The entire amino acid sequence of aurelin (AACSDRAHGHICESFKSFCKDSGRNGVKLRANCKKTCGLC) is not homologous with any known antimicrobial peptides. It contains six cysteines linked by three disulfide bonds. Aurelin has activity against Gram-negative bacteria and Gram-positive bacteria (Ovchinnikova et al., 2006).

### Tunicata

The mostly studied tunicates are ascidians, and many antimicrobial peptides have been isolated from various hemocytes. According to their primary structure, the antimicrobial peptides family can be divided into several categories, which are described below.

#### Styelins

**FIGURE 2:**
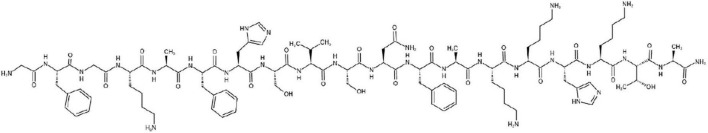
The chemical structure of Styelin A.

Styelins are phenylalanine-rich antimicrobial peptides which were purified from the hemocytes of the tunicate *Styela clava*. The peptides have similar masses (Styelin A, 3685.8; Styelin B, 3700.6) and amino acid compositions (Figure 2). Reportedly, at least 17 of their first 20 N-terminal residues are identical (Lee et al., 1997a).

Styelin A- G X FG K AF X SV SNF A KKHKTA

Styelin B- G X FG P AF H SV SNF A KKHKTA

Both styelins were effective against a panel of Gram-negative and Gram-positive bacterial pathogens of humans (Lee et al., 1997a). They usually act at minimal inhibitory concentrations of <1.5 μg/mL (<0.5 μM), even when 100 mM NaCl is present. They also eliminated the marine bacteria *Psychrobacter immobilis* and *Planococcus citreus* in media containing 0.4 M NaCl. Styelins in tunicate hemocytes can be seen as evidence that such molecules are very old mediators of the host defense system. Peptide antibiotics from marine organisms could afford a design template for the development of topical microbicides that can manifest broad-spectrum antibacterial activity in the presence of physiological or elevated NaCl concentrations (Taylor et al., 2000).

#### Clavanins and Clavaspirin

These two peptides are amphiphilic α-helical peptides. They were isolated from hemocytes of the invertebrate *Styela clava*, which is native to the Pacific coast of Asia, ranging from the Sea of Okhotsk to Japan, South Korea, and northeast China (Lee et al., 1997b). Each member (A, B, C, and D) is C-terminally amidated and they all have 23 amino acid residues (Lehrer et al., 2001). Clavanin A is unusually rich in phenylalanine, with 5 out of 23 residues, which suggests that these residues are functionally important (van Kan et al., 2003a,b). Clavanin A plays its role at the molecular level of the membrane via a pH-dependent mechanism. At a neutral pH, clavanin disrupts biological membranes in a non-specific manner, causing an efflux of large molecules. At mildly acidic conditions, the peptide effectively kills bacteria by permeabilizing their membrane (van Kan et al., 2002). In addition, clavanins are generally active against Gram-positive bacteria, including methicillin-resistant *Staphylococcus aureus*. Clavanin A is an antimicrobial peptide that could possibly augment the development of novel peptide-based strategies to help treat sepsis and wound infections (Silva et al., 2015).

Clavanin A: V F QF LG K IIH HVGNFV H GFS HVF

Clavanin B: V F QF LG R IIH HVGNFV H GFS HVF

Clavanin C: V F HL LG K IIH HVGNFV Y GFS HVF

Clavanin D: A F KL LG R IIH HVGNFV Y GFS HVF

Clavaspirin: FLRFIGSVIHGIGHLVHHIGVAL-NH2.

Clavaspirin is a histidine-rich amidated 23-residue peptide that is very close to the clavanins. It was isolated from pharyngeal tissues of the tunicate *Styela clava* (Lee et al., 2001a). Synthetic clavaspirin can kill Gram-positive and Gram-negative bacteria. It infiltrates into the outer membrane and inner membrane of *E. coli* and then dissolves the phosphatidylglycerides. This peptide can also induce erythrocytolysis in human and bovine subjects (Lee et al., 2001a). Although clavanins and clavaspirin have a few similarities in their primary structures, the signal peptides and cationic extension regions of the precursors of these two antimicrobial peptides have a high degree of homology in nucleotide and amino acid sequences (the sequence similarity is greater than 90%). Both contain a signal peptide of 19 residues, a propeptide of 10 residues, a mature peptide of 24 residues and a posterior peptide of 27 residues, and both the propeptide and the posterior peptide contain cations.

#### Plicatamide and Halocyamines

Plicatamide (Phe-Phe-His-Leu-His-Phe-His-dc Delta DOPA, where dc Delta DOPA represents decarboxy-(E)-α, β-dehydro-3,4-dihydroxyphenylalanine) is a potently antimicrobial octapeptide from the hemocytes of the solitary tunicate *Styela plicata*. Plicatamide causes a huge drain of K^+^ from wild-type and methicillin-resistant *Staphylococcus aureus*; it can also dissolve human erythrocytes. Plicatamide shows slightly positive electrical activity when the pH is 7.4, indicating its optimal antimicrobial activity (Tincu et al., 2003).

Halocyamines (L-3,4-dihydroxyphenylalanine Delta DOPA, bromoindole) are tetrapeptides taken from the morula cells of the solitary ascidian *Halocynthia roretzi*. Halocyamine has two isomers, namely Halocyamine A and Halocyamine B. Their structures were determined to be L-histidyl-L-6, 7-dihydroxyphenylalanylglycyl-6-bromo-8, 9-didehydrotryptamine and L-threonyl-L-6, 7-dihydroxyphenylalanyl-L-histidyl-6-bromo-8, 9-didehydrotryptamine, respectively. In addition to demonstrating antibacterial activity against *Saccharomycetes* and some marine bacteria (*Achromobacter aquamarinus* and *Pseudomonas perfectomarinus*), it is cytotoxic to cultured rat embryonic brain neuron cells, mouse neuroblastoma N-18 cells and human hepatoma Hep-G2 cells (Azumi et al., 1990).

#### Dicynthaurin and Halocidin

Dicynthaurin was isolated from hemocytes of a tunicate, *Halocynthia aurantium*. The native peptide had a mass of approximately 6.2 kDa and comprised two 30-residue peptides (ILQKAVLDCLKAAGSSLSKAAITAIYNKIT) (Lee et al., 2001b). It showed antibacterial activity against *Micrococcus luteus, Staphylococcus aureus*, *Listeria*, *Escherichia coli*, and *Pseudomonas aeruginosa*, but not *Candida albicans* (Jang et al., 2005). However, its best antibacterial condition is when the salinity is lower than 100 mmol/L, which implies that this molecule may have an antibacterial effect in cells such as phagosomes, not outside a cell (Lee et al., 2001b).

Halocidin, with a molecular weight of about 3.44 kDa, was also isolated from hemocytes of a tunicate, *Halocynthia aurantium.* It is a dimer formed by connecting two peptides composed of 18 amino acids (WLNALLHHGLNCAKGVLA) and 15 amino acids (ALLHHGLNCAKGVLA) through disulfide bonds. Halocidin has strong antibacterial activity against *Staphylococcus aureus* and *Pseudomonas aeruginosa*. Its 18 residues and the dimer composed of 18 residues have mild hemolytic activity against human red blood cells (Jang et al., 2002).

The antimicrobial peptides families and their features discussed above have been summarized in Table 1.

## Mechanisms of Action

Since the discovery of antibacterial peptides, much research has been conducted on their mechanism. Antimicrobial peptides may act on bacteria in a variety of ways, leading to lysis and death. Antibacterial peptides and antibiotics have different antibacterial mechanisms. The former mainly include the attraction, attachment, insertion and orientation of peptides (Montero-Alejo et al., 2017). Earlier, it was reported that positively charged antimicrobial peptides combined with negatively charged phospholipids in cell membranes are the only bactericidal mechanism. Recently, more studies have shown that antimicrobial peptides can directly kill pathogens by binding to non-membrane targets of the cell walls or intracellular components (Moravej et al., 2018). In addition, antimicrobial peptides can also participate in immune regulation in the body and exert antibacterial effects indirectly (Shahmiri et al., 2015).

Interestingly, one common feature of the antibacterial effects of antimicrobial peptides is that they must first approach or touch the surface of bacterial cells before they can take effect. After being combined with bacteria, the bacteria-killing effect of peptides mainly depends on insertion into the bacterial cell membrane. This will cause leakage of the intracellular material or destroy the transmembrane’s potential, which causes abnormal physiological processes and cell death (Shai, 1999; Huang, 2000).

### Membrane-Targeting Mechanism

**FIGURE 3:**
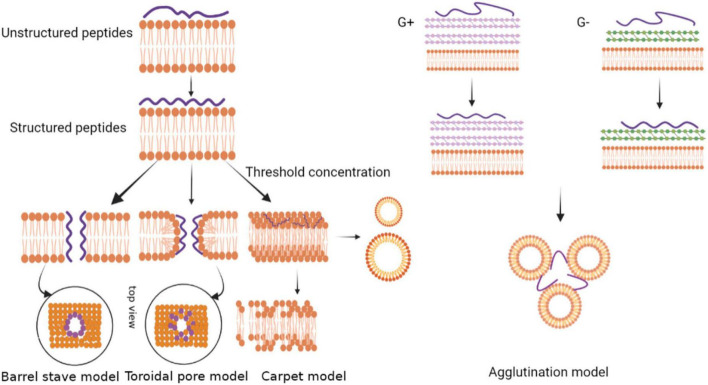
Models depicting the mode of action by membrane-active antimicrobial peptides.

The electrostatic binding of antimicrobial peptides to cell membranes is one of the primary and critical steps of killing the invading pathogens. The initial driving force of peptide molecules against pathogens is the electrostatic attraction due to the opposing charge between the antimicrobial peptide and the membrane. In addition to anionic lipids, lipopolysaccharides (LPS) and lipoteichoic acids on the surface of the bacterial outer membrane also drive targeting electrostatically (Kang et al., 2017). After the antimicrobial peptides target and bind to the outer membrane of the pathogen, some of the antimicrobial peptides diffuse through the outer membrane to the inner membrane. Since the binding is driven by electrostatic interactions, the degree of penetration into the intima increases as more antimicrobial peptide molecules arrive. The antimicrobial peptides at the endometrial target reach a concentration threshold or a minimum peptide concentration for antimicrobial activity (Ciumac et al., 2019). During experiments, the threshold concentration is usually expressed as the peptide/lipid ratio (P/L). The peptide to lipid ratio determines the properties of the AMPs’ insertion membrane. At a high peptide-to-lipid ratio, the antimicrobial peptides are vertically oriented and form transmembrane pores. At a low peptide to lipid ratio, the antimicrobial peptides are inserted parallel to the bacterial membrane (Sierra et al., 2017). In general, amphoteric antimicrobial peptides play an antimicrobial role by interacting with cell membranes due to hydrophobic and electrostatic reactions (Tossi et al., 2000). Clavanins, for example, exert their antimicrobial activity mainly through infiltrating the microbial target cell membrane (Van Kan et al., 2001). There are four membrane targeting mechanism modes, namely the carpet model, the barrel-stave model, the toroidal pore model and the agglutination model (Figure 3).

#### Carpet Model

On the surface of an antimicrobial peptide, the positive charge is combined with the anionic phospholipid head through an electrostatic interaction, and the peptide is bound to the membrane in a parallel direction (Moravej et al., 2018). After accumulation of the peptides, a carpet-like structure is formed on the phospholipid membrane to cover the surface of the microbial cell membrane. The high concentration of antimicrobial peptides interacts with phospholipid head clusters outside the membrane to cause membrane permeation (Lee et al., 2019). The peptide forms a variety of instantaneous pores on the membrane at a certain threshold concentration, acting in a detergent-like manner to form micelles, and finally cell lysis occurs (Zhang and Gong, 2020). The mechanism of the antimicrobial peptide perinerin can be explained by this model (Shai, 1999).

#### Barrel-Stave Model

In amphiphilic antimicrobial peptides, the hydrophobic region first aligns with the phospholipid head on the membrane surface, and then reaggregates into a barrel-like channel perpendicular to the membrane surface. Meanwhile, the positively charged hydrophilic region aggregates to form the inner wall of the channel (Yang et al., 2001). Monomer peptides form aggregates in an α-helical structure on the membrane surface. The aggregates are further inserted into the membrane. Therefore, their non-polar side chains point to the hydrophobic core of the membrane, while the hydrophilic surface of the peptide points to the inner side of the membrane. The formation of transmembrane pores causes intracellular substances to leak out, which leads to cell death (Shabir et al., 2018).

#### Toroidal Pore Model

The toroidal pore model is a model between the barrel-stave model and the carpet model. Antimicrobial peptides form an α-helix on the surface of the cell membrane and are inserted vertically into the lipid bilayer. They then make the lobules in the bilayer to bend and form transient pores (Wang et al., 2017). The peptide chain is embedded in the hydrophilic and hydrophobic interface, and the lipid bilayer head is arranged inside the pore. The difference between the toroidal pore model and the barrel-stave model is that the peptides in the toroidal pore model are always associated with the polar membrane groups even after the hole has formed, while the peptides in the barrel-stave model are separated immediately after the formation of the central cavity (Sharma et al., 2018). Relevant studies have shown that antimicrobial peptides that target intracellular components may enter the cell through toroidal pores, then the transient pores destroy the barrier function of the membrane and cause the loss of transmembrane potential (Ciumac et al., 2019). However, only a few peptides exert antibacterial effects through this mode of action. Among the cnidarians, hydralysin, a paralytic toxin isolated from *Chlorohydra viridissima*, mainly exists in solution as a β-structure, which can form pores with a diameter of 1.2 nm on the erythrocyte membrane of pathogens (Sher et al., 2005).

#### Agglutination Model

The agglutination model is a model that is completely different from pore formation in the membrane. The agglutination model involves a micellar complex formed by the combination of cationic peptides and outer membrane lipopolysaccharides of Gram-negative bacteria or cell wall peptidoglycans of Gram-positive bacteria. This induces bacterial cell aggregation, and the peptides do not penetrate the cell membrane. Agglutinated cells are easily swallowed and so they prevent the release of toxic substances (Sinha et al., 2017). A cationic anti-LPS factor was found in the hemolysis products of *Tachypleus tridentatus* and *Limulus polyphemus*. The anti-LPS factor could inhibit hemolymph agglutination in horseshoe crab and has a strong inhibitory effect on the growth of Gram-negative bacteria (Muta et al., 1987). Antimicrobial peptides from the horseshoe crab are released at the infected site of microorganisms. The peptides combine with the bacterial LPS to form a complex and produce anti-LPS factors that inhibit bacterial growth (Hirakura et al., 2002).

In short, the membrane-targeting mechanism of antimicrobial peptides can be described as a variety of action mode, and different antimicrobial peptides can produce bactericidal effects via these different action models. It should be noted that traditional antibiotics target specific proteins on the surface of microorganisms, while antimicrobial peptides can target the entire surface of cell membranes. Thus antimicrobial peptides make it difficult for bacteria to develop resistance through mutation, which is of great significance for the development of new drugs.

### Non-membrane-Targeting Mechanisms

The mechanisms of membrane permeation were proven to be the main mode of action of antimicrobial peptides. However, some other mechanisms have also been proposed and widely discussed. For example, in the non-membrane targeting mechanism mode, antimicrobial peptides inhibit the synthesis of intracellular or extracellular proteins, nucleic acids, lipoteichoic acid, peptidoglycan and other biopolymers. Thereby, cell wall synthesis becomes abnormal, leading to abnormal metabolism and cell death (Zhang et al., 2019). Studies have shown that besides the cell wall, antimicrobial peptides can directly act on nucleic acids after entering cells to affect the replication and synthesis of DNA and RNA. Peptides also act directly on protein molecules. They change the structure and inactivate the protein, ultimately leading to cell death (van der Does et al., 2019). In addition, antimicrobial peptides also work by blocking energy transfer. Antibacterial peptides can inhibit ATP synthesis by disrupting the activity of ATP synthase or blocking the electron transport chain, resulting in impaired energy metabolism. At the same time, antimicrobial peptides reduce the activity of ATP-dependent enzymes by directly interacting with ATP. Due to the impaired energy metabolism or reduced enzyme activity, intracellular biological processes dependent on ATP are blocked, leading to cell death.

Apparently, the cell membrane is not the only target and antimicrobial peptides also act on cell walls, intracellular nucleic acids, proteins, enzymes, and organelles. After binding to the targets, they inhibit key intracellular processes, obstructing the pathogen’s metabolism and eventually causing the death of the bacteria. Research into the mechanism of the intracellular targets of antimicrobial peptides can prevent the pathogen from developing resistance to them.

### Immune Regulation Mechanism

In addition to killing pathogens directly, antimicrobial peptides can also exert their effects through immune regulation mechanisms. Antibacterial peptides are mainly produced by secretion from immune cells, which can produce a variety of immune responses. The peptides are involved in the differentiation of relevant immune cells and activation of immune cells (macrophages, monocytes, dendritic cells, and T cells). Furthermore, they neutralize the pro-inflammatory cytokines released from macrophages and monocytes, inhibiting inflammation (Kumar et al., 2018). Studies have shown that antimicrobial peptides may also interfere with normal cell functions, such as gene transcription, apoptosis and cytokine production, so that the host’s innate immunity and adaptive immunity are enhanced (AlMatar et al., 2018).

In summary, antimicrobial peptides have a variety of mechanisms of action and a rich range of targets, which makes it difficult for pathogens to develop drug resistance, greatly reducing the risk of use. It is expected that a new generation of antimicrobial drugs will replace antibiotics.

## Conclusion

Antimicrobial peptides are widely found in various animals and plants and are the first line of defense against pathogens. Antimicrobial peptides have marked cationic and amphipathic characteristics. Their cationic property helps in the initial binding of the peptides to the pathogen, and their amphiphilic nature helps them to bind to the bacterial membranes and disintegrate them. As a new type of small-molecule “antibiotic,” antimicrobial peptides have broad spectrum activities, high potency, richly available natural sources, particularly good safety and tolerance, and other advantages that traditional antibiotics do not have. They have therefore attracted increasing attention from researchers. In recent years, great progress has been made in research into antimicrobial peptides. Antimicrobial peptides not only have antibacterial, antiviral, antitumor and antiparasitic activities, but they also have immunomodulatory, antioxidant and anti-inflammatory activities. Therefore, some natural peptides have been widely applied in many fields, especially in the medical field. Because antimicrobial peptides can neutralize endotoxins, they can be used to treat sepsis. There is also increasing evidence that antimicrobial peptides can inhibit the inflammatory response and avoid an excessive inflammatory response in regulating the vertebrate immune response.

With the deepening of research on antimicrobial peptides from marine invertebrates, there are still many problems regarding how to better utilize and apply the activity of antimicrobial peptides. Marine invertebrates are diverse in species and large in number, so it is difficult to obtain antimicrobial peptide samples. Marine invertebrates only produce a small amount of antimicrobial peptides, which makes it difficult to find, isolate and identify antimicrobial peptides. At the same time, it is necessary to develop and apply biological analysis methods with high sensitivity, high accuracy and high selectivity, alongside high-throughput isolation and identification methods to promote the discovery of antimicrobial peptides. Some studies have also shown that many specific antimicrobial peptides isolated thus far are cytotoxic and can cause hemolysis in human erythrocytes. They are easily degraded by proteases because of the small size.

Many problems still face research into antimicrobial peptides, such as technical obstacles, high cost, biodegradation and adverse pharmacokinetics. These problems will be gradually solved with the deepening of research and technological progress. As pathogenic microorganisms continue to spread and create issues for typical and traditional antibiotics, creating new solutions has become an urgent matter that requires immediate action. Antimicrobial peptides not only have multiple mechanisms of action and rich targets, making it difficult for pathogens to develop drug resistance and greatly reducing the risk of use, but also have a small size and a broad spectrum. There is no doubt that natural peptides are a good candidate for exploring antimicrobial resistance. It is expected that a new generation of antimicrobial drugs will replace antibiotics.

## Author Contributions

RW, JP, EN, and PO prepared the original draft. MV, WW, and KK upgraded the original draft and finished the manuscript. All authors contributed to the article and approved the submitted version.

## Conflict of Interest

The authors declare that the research was conducted in the absence of any commercial or financial relationships that could be construed as a potential conflict of interest.

## Publisher’s Note

All claims expressed in this article are solely those of the authors and do not necessarily represent those of their affiliated organizations, or those of the publisher, the editors and the reviewers. Any product that may be evaluated in this article, or claim that may be made by its manufacturer, is not guaranteed or endorsed by the publisher.
